# The impact of genetic background and cell lineage on the level and pattern of gene expression in position effect variegation

**DOI:** 10.1186/s13072-019-0314-5

**Published:** 2019-11-13

**Authors:** Sidney H. Wang, Sarah C. R. Elgin

**Affiliations:** 1 Center for Human Genetics, The Brown Foundation Institute of Molecular Medicine, The University of Texas Health Science Center at Houston, Houston, TX USA; 2 Department of Biology, Washington University, St Louis, MO 63130 USA

**Keywords:** PEV, Heterochromatin, Modifiers of PEV, Transcription regulation

## Abstract

**Background:**

Chromatin-based transcriptional silencing is often described as a stochastic process, largely because of the mosaic expression observed in position effect variegation (PEV), where a euchromatic reporter gene is silenced in some cells as a consequence of juxtaposition with heterochromatin. High levels of variation in PEV phenotypes are commonly observed in reporter stocks. To ascertain whether background mutations are the major contributors to this variation, we asked how much of the variation is determined by genetic variants segregating in the population, examining both the level and pattern of expression using the fruit fly, *Drosophila melanogaster*, as the model.

**Results:**

Using selective breeding of a fourth chromosome PEV reporter line, 39C-12, we isolated two inbred lines exhibiting contrasting degrees of variegation (A1: low expression, D1: high expression). Within each inbred population, remarkable similarity is observed in the degree of variegation: 90% of the variation between the two inbred lines in the degree of silencing can be explained by genotype. Further analyses suggest that this result reflects the combined effect of multiple independent *trans*-acting loci. While the initial observations are based on a PEV phenotype scored in the fly eye (*hsp70*-*white* reporter), similar degrees of silencing were observed using a *beta*-*gal* reporter scored across the whole fly. Further, the pattern of variegation becomes almost identical within each inbred line; significant pigment enrichment in the same quadrant of the eye was found for both A1 and D1 lines despite different degrees of expression.

**Conclusions:**

The results indicate that background genetic variants play the major role in determining the variable degrees of PEV commonly observed in laboratory stocks. Interestingly, not only does the degree of variegation become consistent in inbred lines, the patterns of variegation also appear similar. Combining these observations with the spreading model for local heterochromatin formation, we propose an augmented stochastic model to describe PEV in which the genetic background drives the overall level of silencing, working with the cell lineage-specific regulatory environment to determine the on/off probability at the reporter locus in each cell. This model acknowledges cell type-specific events in the context of broader genetic impacts on heterochromatin formation.

## Background

Position effect variegation (PEV) describes the mosaic expression of a phenotype in a cell population that is otherwise thought to be uniform. It has generally been studied in cases where the cell-autonomous phenotype is easily visualized, such as eye pigmentation, but appears to be a general phenomenon [1, 2]. Muller reported the original observation of variegating eye pigmentation in adult flies, recovered following X-ray mutagenesis. Because of the high degree of variation in the pattern, and in the level of pigmentation between individuals and across generations, he described the phenotype as “ever sporting” [3]. The report on this highly variable phenotype led to various speculative models describing how such a heritable, yet variable phenotype could arise [4]. Further investigations have led to a generally accepted transcriptional silencing model based on a stochastic spreading of heterochromatin [5]. The X-ray-induced inversion generated by Muller juxtaposed the *white* gene, which is required cell autonomously for proper deposition of eye pigment, with the pericentric heterochromatin. The spreading of pericentric heterochromatin packaging to the *white* locus results in concomitant silencing; when this occurs in some but not all of the cells, the result is a variegated pattern of eye pigmentation. This spreading process is thought to be stochastic (reviewed in [6]).

The key concept that enables the spreading model to describe a variegating phenotype is the implicitly assigned probability of heterochromatin spreading. It is intuitive to consider that a locus closer to the pericentric heterochromatin would be more likely to be silenced by the spreading of heterochromatin than a locus that is further away; thus, an inversion (or transposition) that brings the *white* locus closer to pericentric heterochromatin would lead to a discernable level of stochastic silencing [5, 6]. Because the process is governed by probability, each cell in a homogenous population could have the same chance of heterochromatin spreading/silencing to a given locus, yet as a whole, different variegating patterns could arise. Many factors have been found to affect the probability of spreading. Genetic mutations are perhaps the best studied. Screens for second-site modifiers of a PEV phenotype have identified numerous loci that have a strong impact on the expression level of the PEV phenotype for the cell population as a whole. These genetic modifiers are referred to as suppressors [Su(var); loss of silencing] or enhancers [E(var); gain in silencing] of PEV (see [2, 6] for review). Some of these loci exhibit antipodal effects, i.e., if one dose of the gene results in loss of silencing, three doses result in an increase in silencing. This antipodal response has been interpreted as evidence that the probability of the heterochromatin spreading process occurring is determined at least in part by the dosage of key gene products that constitute the structural components of heterochromatin; in other cases, an enzymatic contribution is implied. An assay scoring a PEV phenotype following a one generation cross to assess dominant effects of candidate PEV modifier alleles has, therefore, been commonly used to test the participation of a given gene of interest in the process of heterochromatin formation and gene silencing [7]. In fact, screens for PEV suppressors have been a major source of candidate genes for participation in the process of heterochromatin formation [8, 9]. Numerous mutations have been identified to modify PEV; it is estimated that there are more than 150 such modifiers in the fly genome [10]. It has long been recognized that genetic background—including different assortment of alleles at these many loci—could affect the probability for a spreading event to occur in a given fly within a stock, and thus could contribute to variation in PEV phenotypes. In fact, a recent study looking at PEV in an outbred fly population suggested that many more modifier loci likely exist across the genome [11].

Although PEV has been tremendously helpful in developing our understanding of heterochromatin, its stochastic nature continues to raise unanswered questions. An arguably more intriguing but much less studied aspect of PEV is the different patterns of variegation. The spreading model effectively describes the variable PEV patterns for classic examples in *S. pombe*, where colonies with sectors of variegating expression are explained by stochastic spreading of heterochromatin followed by clonal inheritance of the chromatin state [1, 12]. In higher eukaryotic systems, however, the effectiveness of the spreading model in describing variegation patterns becomes much less clear and possibly locus dependent. Compared to *S. pombe,* where individual cells in a population are often considered identical, in multi-cellular organisms, cells differentiate, and it becomes much less clear how often a population of cells can be considered effectively homogenous for a locus of interest.

We previously devised a P element reporter to probe the heterochromatin landscape of the genome, P{*hsp26*-*pt, hsp70*-*white*}. Using the well-characterized *hsp70* promoter to drive a *white* reporter gene, about 1% of the insertion lines recovered following mobilization exhibit a variegating eye phenotype [13]. Mapping of these variegating insertion lines revealed an outline of heterochromatin distribution in the genome, which is in agreement with prior cytological assignments, but provides higher resolution. PEV is observed following insertion of the reporter P element into the pericentric and telomeric regions of the major autosomes and the X chromosome, as well as regions of the Y chromosome. Based on the eye phenotype, the fourth chromosome (Muller F element), while largely heterochromatic, appears to have interspersed heterochromatic and permissive domains [14]. Characterization of these variegating P element reporter insertion lines has indicated that the basic principles for variegation as observed in the original *white* mottled line from Muller (i.e., sensitivity to sex chromosome dosage, etc.) are common to most variegating lines [13], although individual heterochromatic domains can show differences in sensitivity to a subset of the known suppressors of variegation [15–17]. A major exception are insertions into the TAS (telomere-associated sequences), just proximal to HeT-A and TART; these lines exhibit a PEV phenotype that is sensitive to mutations in the Polycomb silencing machinery [18], while ChIP analysis shows association with Pc [19].

Individual flies from each of the laboratory stocks of P{*hsp26*-*pt, hsp70*-*white*} reporter lines often show a high level of variation in the degree of variegation; in contrast, there is often a discernable similarity in the pattern of variegation for a given stock. Both observations are in clear contradiction to what would have been predicted by the spreading model per se; assuming homogeneous cell populations in a uniform genetic background, the spreading model predicts uniform degrees of PEV without a consistent pattern between individual flies. We anticipate that background genetic variants contribute to the variable degrees of PEV commonly observed between individuals in these reporter lines, and that for certain reporter lines, the homogeneous cell population assumption is inadequate. Our goal is to generate quantitative evidence addressing this hypothesis. Here we have used as our test locus a reporter in the fourth chromosome, a largely heterochromatic domain that for the most part mimics pericentric heterochromatin, a chromatin structure dependent on H3K9 methylation and associated HP1a [13, 15]. The study was carried out using a 4th chromosome PEV reporter line, 39C-12, for several reasons. 39C-12 is relatively well characterized in terms of its response to PEV modifiers [15, 16, 20]. Its position has been mapped to a precise location in the genome [14]. The *hsp70* promoter used in this reporter is well characterized [21]. Its basal activity at 25 °C in this construct is sufficient to cause a uniform red eye when the reporter is inserted into a euchromatic site. Finally, the 39C-12 stock is considered relatively “clean” because of the genetic bottleneck that occurred during the production of the transgenic line; specifically, the line is derived from a single male with the P element insertion on the fourth chromosome, back-crossed to *yw*^*67c23*^ females. Taking a quantitative genetics approach, we found that most of the variation in the degree of PEV in the 39C-12 stock could be explained by genetic variants floating in the background, likely a combination of residual heterozygosity from backcrossing and new mutations accumulated over time (ca. 15 years in stock). This result was duplicated with a second reporter juxtaposed to Y heterochromatin using a different crossing scheme. In addition, we formally tested pattern enrichment for 39C-12 PEV and found significant enrichment at the ventral-posterior quadrant of the fly eye, supporting anecdotal observations of similarity between individual flies in reporter stocks. Similar patterns in PEV phenotype among individual flies indicate consistent differences between cells in their ability to silence the reporter. Our observations are consistent with the published literature and provide fresh insights into this classic system.

## Results

**Fig. 1:**
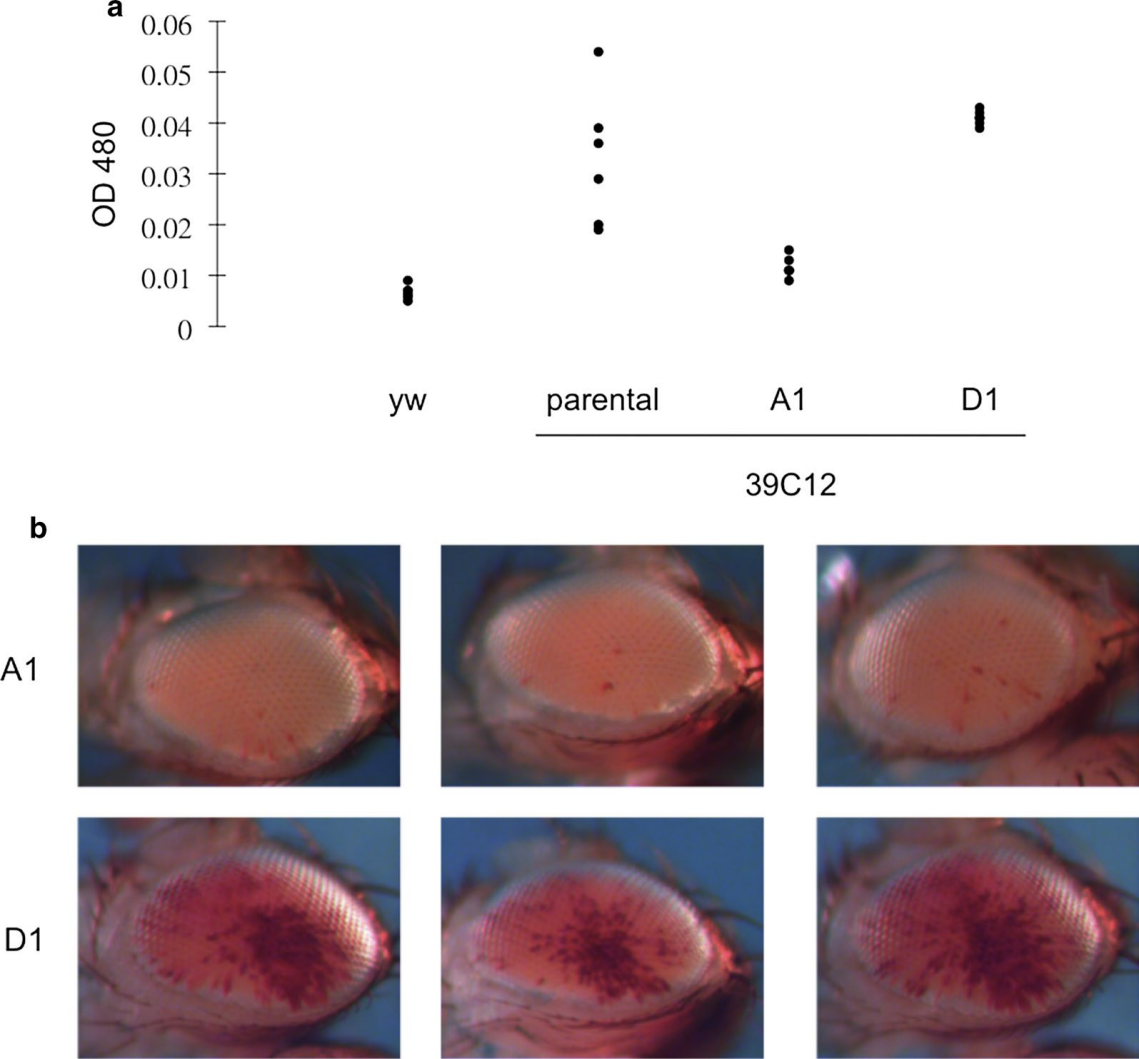
Selective inbreeding results in highly consistent PEV phenotypes within a laboratory population. **a** Quantitative assessment of pigment levels in the adult fly eye representing the degree of PEV. Each data point represents a reading from samples of five flies from a population of the indicated genotype, parental (39C-12) or selected (A1, D1) (see “Materials and methods” for details). *yw* is used to indicate the background pigment level. **b** Images of the PEV pattern in the adult fly eye taken from randomly selected individuals in each of the A1 and D1 inbred populations

Visual inspection shows considerable variation in the levels of PEV in adult fly eyes among individuals of the 39C-12 reporter line raised at 25 °C. Despite a genetic bottleneck during the production of this transgenic line, there is a high degree of variation in the level of extracted eye pigment (coefficient of variation [CV] = 51.3%), which averaged 0.0246 (OD_480_). Because the reporter line was created in the 90s, we hypothesize that mutations accumulated over time on top of the residual heterozygosity in the starting stock could have contributed, at least in part, to the phenotypic variation observed. To study the underlying genetic contribution to the variation of PEV among individual flies, we first selected for extreme PEV phenotypes (based on eye pigment levels) by selective breeding. A single virgin female from the parental population displaying the strongest PEV eye phenotype (the least pigment) was mated to a single male sibling with a matching eye phenotype. This process was repeated for five generations (i.e., full sibling mating followed by selection) to obtain a fly line, 39C-12-A1, in which the level of eye pigmentation is lower (i.e., strong PEV; mean = 0.0104; SD = 0.0020) and more consistent among individuals (CV = 19.23%; Fig. 1; Table 1) than the starting population. A weak PEV line, 39C-12-D1, was similarly derived (Fig. 1, Table 1; mean = 0.0345; SD = 0.0076; CV = 22.03%). The two inbred lines have a 3.3-fold difference in pigment level (*p* < 1e−11, ANOVA) and represent the extreme ends of the phenotypic spectrum of the original population (i.e., each is about one standard deviation away from the mean of the original 39C-12 population, in opposite directions). In addition to the genetic effect introduced by selective breeding, there is also a sex effect impacting the PEV phenotype. While there is a modest 26.02% higher pigment level in A1 males relative to A1 females (*p* < 0.05), there is a 47.93% higher pigment level in D1 males compare to D1 females (*p* < 0.001). Overall, combining genotype and sex effects in a linear model explains 95.9% of the variance between A1 and D1 flies, while genotype alone explains 89.6% of the variance (see “Materials and methods” and “Statistical analysis”). In other words, the effect of selective breeding contributes to the majority of the phenotypic differences observed between the two inbred lines, supporting the hypothesis that background genetic variants are the major contributors to the phenotypic variation observed in the parental 39C-12 population.

**Table 1:** Pigment assay results for 39C-12 inbred variegating lines

	Mean (OD_480_)^a^	Coefficient of variation (%)	Sample size
Starting stock	0.0246	51.30	12
A1	0.0104	19.23	12
D1	0.0345	22.03	12
F1	0.0196	14.02	12
F2	0.0216	51.56	10

^a^Average values reported for measurements of pigment level; each measurement was obtained from pigment extracted from a pool of five representative flies as one sample

**Fig. 2:**
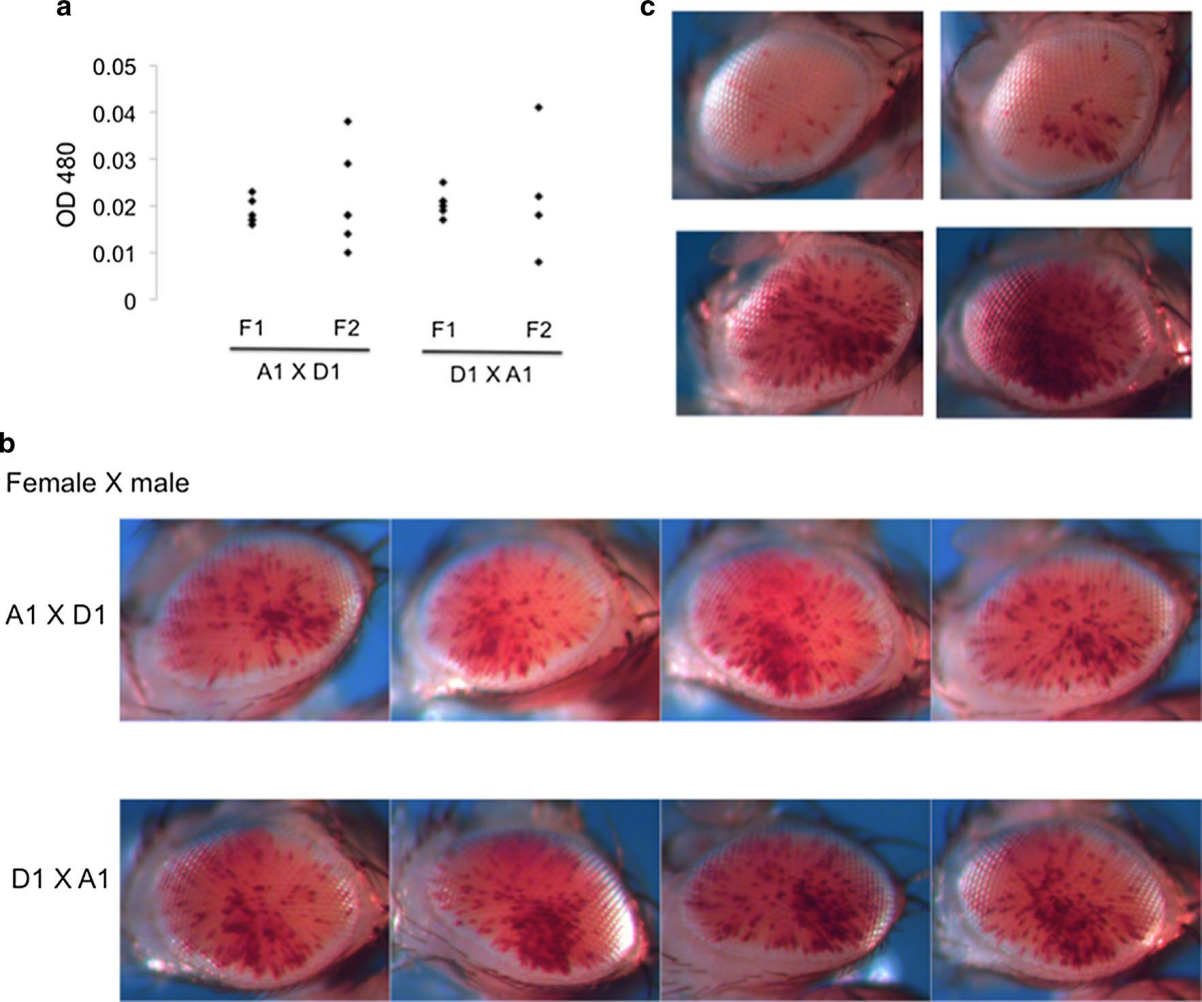
PEV phenotype of the progeny from the cross between the A1 and D1 inbred lines. **a** PEV levels in the adult progeny. Each data point represents a sample of five flies from a population of the indicated genotype (see “Materials and methods”). Results observed were essentially the same from crosses in either direction (females listed first). **b** The PEV pattern in the adult fly eye from randomly selected F1 progeny of a cross between the A1 and D1 inbred lines in the indicated direction. **c** Selected images of the PEV pattern in the F2 population representative of the diversity in pigmentation levels observed

Mutations accumulated over time are expected to broadly distribute across the genome. To assess the genetic architecture (i.e., the underlying genetic basis of the phenotypic differences [22]) of the two inbred lines regarding the impact on the PEV phenotype, crosses were performed between these lines to generate F1 and F2 populations. There is a fairly consistent intermediate PEV phenotype in the F1 population (mean = 0.0196, SD = 0.0027, CV = 14.02%). Similar results were obtained from crosses in both directions (Fig. 2a, b). The average pigmentation level for F1 progeny in both cases falls right in the middle between the pigment levels of the parental A1 and D1 lines (Figs. 1a, 2a; Table 1). As would be expected for a quantitative trait involving multiple independent loci, a wide spectrum of PEV phenotypes was observed in the F2 population (mean = 0.0216, SD = 0.0111, CV = 51.56%), which likely resulted from meiotic recombination and random segregation of the A1 and D1 background PEV modifiers. The mean pigmentation level for the F2 progeny is similar to the F1 population (0.0216 vs. 0.0196); in contrast, there is a large increase in the range of expression levels for the PEV phenotype between individuals of the F2 population (CV = 51.56% vs. 14.02%; Fig. 2a, c, Table 1). The range of phenotypic variation in the F2 population resembles that of the starting 39C-12 stock (compare Fig. 1a with Fig. 2a, CV = 51.3% vs. 51.56%). Note that the differences observed in CV are not a spurious observation driven by a few outliers; further analysis confirmed the robustness of the result (Additional file 1: Figure S1). Taken together, these results suggest that the variation in PEV phenotype between individuals of the 39C-12 transgenic fly line is best described by the effect of multiple *trans*-genetic modifier loci acting independently in the background, which further supports the background mutation hypothesis.

**Fig. 3:**
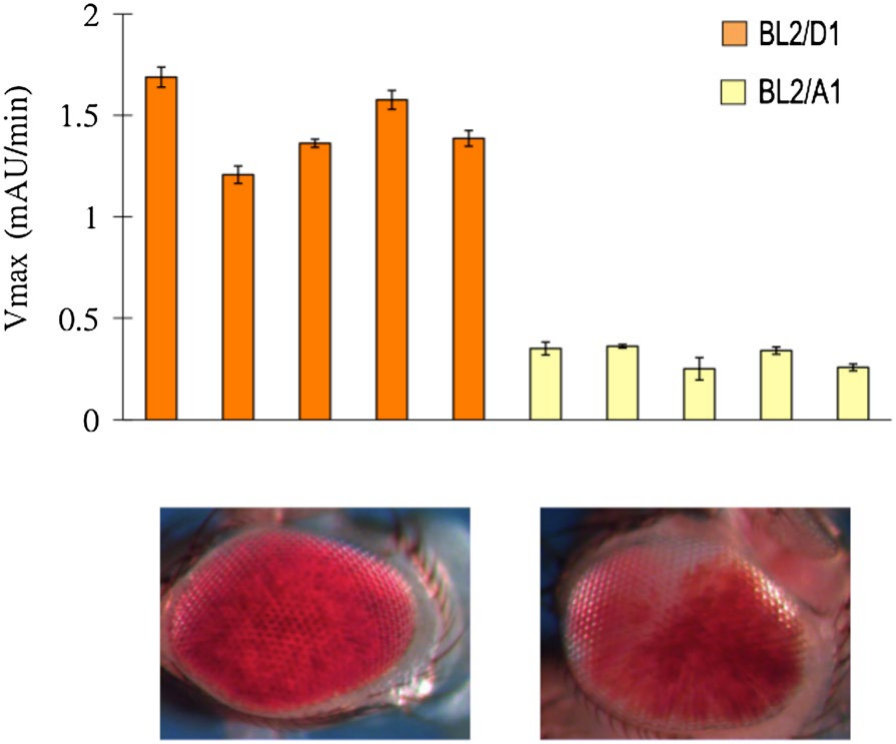
PEV phenotype of the Y-linked BL2 reporter in the A1 and D1 genetic backgrounds. The level of PEV is quantified by measuring the activity of the *beta*-galactosidase reporter gene. Each bar represents the activity level measured in lysate prepared from one adult whole fly of the indicated genotype. Bar height and error range represents the mean and standard error calculated from technical replications (i.e., measurements made on aliquots of the same lysate). Representative images of eye pigmentation for each genotype, shown below the bar graph, show the variegating phenotype anticipated

The results above are based on the PEV eye phenotype of a P element *hsp70*-*white* reporter inserted into the heterochromatic 4th chromosome. To determine whether the conclusions drawn are generally applicable to the PEV phenotype, we evaluated the impact of the A1 and D1 background genotypes on the PEV phenotype of a Y-linked *hsp70*-*LacZ* PEV reporter, *Tp(3;Y)BL2* (BL2). The PEV phenotype of the BL2 *LacZ* reporter line used for this purpose results from a translocation of a fragment of the 3rd chromosome carrying the reporter to the Y chromosome following X-ray irradiation [23]. A multigeneration cross-scheme was designed to introduce this Y-linked PEV reporter into the A1 (or D1) genotype, without perturbing that genetic background, utilizing dominantly marked balancer chromosomes and relying on the fact that meiotic recombination is not known to occur in the male germ line [24, 25] (Additional file 2: Figure S2). Two inbred lines containing the Y-linked BL2 reporter in the A1 and D1 genetic backgrounds, respectively, were derived (BL2-A1 and BL2-D1). The level of *beta*-galactosidase activity (mAU/min) in lysates prepared from single male flies was used as a quantitative readout for the PEV phenotype. A consistent PEV phenotype for the BL2 reporter across individuals from each of the A1 or D1 genetic backgrounds was observed, with D1 flies exhibiting ~ 4.64 times the activity of A1 flies (Fig. 3). The BL2 reporter in a D1 background gave a CV of 13.11% (mean = 1.44, SD = 0.19), while in an A1 background it gave a CV of 17.24% (mean = 0.31, SD = 0.05). Taken together, the results for the BL2 reporter largely recapitulate the results for the 39C-12 reporter, indicating that the same (or similar) background PEV modifiers impact both a 4th chromosome P element PEV reporter and an X-ray induced Y-chromosome-linked PEV reporter in the same direction. Lu et al. [26] reported variegating expression for the BL2 reporter in multiple tissues, such as various differentiating imaginal discs in late third instar larvae, as well as in adult eyes. Here, BL2 PEV was surveyed using the whole fly in a quantitative assay. Given Lu et al. ’s results, the findings here generalize the impact of background modifiers on PEV (i.e., heterochromatic silencing) beyond the fly eyes analyzed using 39C-12. It is noteworthy that the insertion of the same P element reporter into different heterochromatic domains results in different PEV phenotypes, with the “salt-and-pepper” pattern commonly associated with insertions into pericentric heterochromatin and the fourth chromosome, while large patch or sectored mosaicism is associated with the Y chromosome [13, 23]. The results above demonstrate similar quantitative responses to PEV modifiers regardless of the overall geometry of the expression pattern.

In its simplest form, the widely accepted model of stochastic heterochromatin spreading assumes a homogeneous cell population (i.e., the same probability of spreading/silencing in each cell). Within an inbred population, the spreading model would, therefore, predict a consistent level of PEV, which, as demonstrated above, is dictated by the genetic background, with highly variable variegating patterns between individuals (i.e., fixed probability but various outcomes). Interestingly, in addition to a consistent level of eye pigmentation, the inbred lines also showed a consistent pattern of eye pigmentation among individuals within each line (Fig. 1b). The apparent consistent pattern observed here would argue that nonrandom variation in heterochromatin silencing among cells plays a role in determining the observed pattern. Similarities in PEV pigmentation patterns have been observed between flies in some of our laboratory stocks; however, those observations were generally anecdotal and had not been carefully evaluated. To formally evaluate similarity in the PEV pattern between individual flies, we ask if certain geographic regions of the fly eye expressed pigmentation more frequently. An enrichment of pigmented ommatidia in a ventral-posterior sector (i.e., near the neck) is observed for both A1 and D1 lines. The pigment enrichment pattern was evaluated by comparing the proportion of ommatidia exhibiting red pigment between the ventral-posterior quadrant of the eye and the rest of the eye (Additional file 3: Figure S3). For the A1 and D1 lines, the ommatidia in the ventral-posterior quadrant are 5.24 times (*p* < 0.05, ANOVA, *n* = 5) and 3.58 times (*p* < 1e−5, ANOVA, *n* = 5), respectively, more likely to exhibit pigment than ommatidia in the rest of the eye (Table 2). Furthermore, in both F1 and F2 progenies of an A1 by D1 cross, where no selection for eye phenotype was done, significant enrichment of the pigment level is also observed in the ventral-posterior quadrant of the fly eye: F1 progeny exhibits a 2.85-fold enrichment (*p* < 1e−6, ANOVA, *n* = 14), while F2 progeny exhibits a 2.72-fold enrichment (*p* < 0.05, ANOVA, *n* = 10) (Table 2). These results indicate differences in the ability of cells in the ventral-posterior quadrant and cells in the rest of the eye to silence the reporter, and point to a need for an augmented stochastic spreading model in which cell-to-cell variation is also considered to properly describe the variegating patterns observed here in inbred lines.

**Table 2:** Image analysis results for 39C-12 inbred variegating lines

	Fold enrichment (i.e., in/out)^a^	Significance	Number of images analyzed
A1	5.24	*p* < 0.05	5
D1	3.58	*p* < 1e−5	5
F1	2.85	*p* < 1e−6	14
F2	2.72	*p* < 0.05	10

^a^Fold enrichment indicates the proportion of pigmented pixels within the ventral-posterior quadrant vs. the proportion of pigmented pixels outside that quadrant

## Discussion

A high level of heterogeneity in PEV phenotypes is commonly observed within laboratory reporter strains, even when variation in the genetic background is reduced (as was the case here) by back crossing to *yw*^*67c23*^. We reasoned that the residual heterozygosity after back crossing and additional mutations accumulated over time could explain some of the variation observed in PEV phenotype between individual flies. Using 39C-12 as a test case, we demonstrated that most of the variation in this PEV phenotype can be explained by genetic variants floating in the background. Our results complement a recent study on PEV phenotype using an outbred population [11]. There, Kelsey and Clark found a large number of genetic variants across the genome significantly associated with the strength of PEV. Here, we have demonstrated that even within a relatively inbred population, one still observes a high level of variation in phenotype, which is best described by the effects from multiple independent modifier loci floating in the background. While this study focused on the genetic background of a single laboratory fly stock, 39C-12, different PEV stocks are likely to have a different combination of background alleles. Given the fact that random mutations are inevitable and the fact that we successfully derived two inbred lines that have more than threefold difference in eye pigment level from a single laboratory population, it is worthwhile considering the implications for using PEV reporter lines for studying chromatin-based transcriptional silencing [2, 6]. In addition to forward genetic screens, novel PEV modifiers are often identified through a reverse genetic screen for dominant effects on PEV using mutant alleles of genes that have been identified or are suspected to play a role in chromatin-based transcriptional silencing (e.g., SETDB1, G9a, PIWI). High levels of variation in the starting reporter PEV line often result in high levels of variation in the readout for the screen; some researchers will, therefore, decide to homogenize the genetic background of the starting reporter line to reduce sampling variation, which will increase their power to detect dominant effects from the mutant alleles. In light of the results presented here, the steps researchers take to homogenize the background could lead them to unwittingly enrich or deplete the genetic background of variants that might interact with the mutant allele of interest and, therefore, amplify or reduce the phenotypic impact. This practice could, therefore, lead to inconsistent findings between labs and potentially misleading results. For more reproducible findings, a simple alternative to increase power would be to increase sample size. One should not, of course, blindly increase sample size in order to reach statistical significance. Another commonly implemented strategy to ensure reproducibility would be to test modifier effects of multiple different alleles of the same gene of interest, ideally alleles generated by different means in different genetic backgrounds.

The results using the BL2 reporter on the Y chromosome reproduced the results observed using the original 4th chromosome P element insertion reporter. This replication is important in three aspects. First, introduction of BL2 to the A1 and D1 backgrounds was done through tracking balancers, a crossing scheme very different from selective breeding. The fact that the results observed were consistent with the hypothesized effects of *trans*-acting background modifiers makes it much harder to interpret the selective breeding results in any other way (e.g., some unknown epigenetic state linked to the reporter locus that follows rules similar to quantitative genetics). Second, the BL2 reporter activity was assayed using whole fly lysate, which extends the observation beyond the fly eye. Third, assuming that these results from one Y chromosome reporter and one 4th chromosome reporter are representative of Y chromosome and 4th chromosome heterochromatin (i.e., given the caveat of small sample size and the limitation that both reporters use a 5′ regulatory region of an *hsp70* gene), the results indicate that the background modifiers in aggregate have similar effects on the PEV phenotype, and by extension, on heterochromatic silencing, of two different chromosomes. This conclusion agrees with many previous studies indicating sharing of individual PEV modifiers (see [6] for review). When considered in conjunction with the mass action model (proposed by Locke et al. and widely accepted [27]), the results are also consistent with a model describing the Y chromosome as a heterochromatic sink that modifies the PEV phenotype of reporters at other genomic loci by trapping structural protein products of PEV modifiers. This effect of Y chromosomes potentially explains the sex-linked impact on PEV observed here and previously reported in many investigations.

However, in contrast to the sharing of modifier effects observed here (and elsewhere) between the Y and the 4th heterochromatin, it is amply documented that different *Su(var)* mutations can have a different impact on reporters in different heterochromatic domains [16, 17, 28]. For example, in Drosophila, the three known H3K9 methyltransferases have different impacts on the pericentric heterochromatin and the fourth chromosome [16]. These published examples of domain-specific effects are reports on individual modifier loci. However, the results are sometimes interpreted as reflections of distinctive heterochromatin domains that have a different composition and/or follow a different set of rules for silencing. It will be worthwhile to evaluate the aggregate effects from a collection of background modifiers, generated using selective breeding based on PEV phenotype of different reporters, on different chromosomal domains (e.g., the effect of A1 background on a 4th chromosome reporter vs. its effect on a pericentric reporter); as differential impacts would support the distinctions inferred. Thus, a future study evaluating the impact of selected genetic backgrounds on multiple reporters inserted in different heterochromatic domains across the genome is likely to reveal new insights into the extent of sharing across domains, helping to define the common features of heterochromatin.

The rather consistent pattern of the eye phenotype became better defined as the flies became increasingly inbred over generations of full sibling crosses. Using the shape of the fly eye and other anatomical structures surrounding the eye as landmarks, enrichment of pigmentation in the ventral-posterior quadrant of the eye was tested. We found significant enrichment for both of the A1 and D1 lines, as well as the F1 and F2 progenies of an A1 by D1 cross. It is not uncommon among fly PEV researchers to observe certain patterns consistently occurring in certain reporter lines. For example, insertions of the *hsp70*-*white* reporter into the Y chromosome often show patterns with large blotches of pigmentation, whereas insertions of the same reporter into pericentric heterochromatin or the fourth chromosome generally results in a fine-scale salt-and-pepper appearance in the eye [13, 28]. However, we are not aware of any prior testing of whether certain geographic locations in the eye are more likely to silence (or fail to silence) the reporter. Here we tested the inbred 39C-12 reporter lines through statistical analysis on images of the PEV phenotype. Significant enrichment of pigmentation in the ventral-posterior sector indicates a lower probability of silencing in that sector relative to the rest of the eye.

The observation of geographical differences in silencing is important in the context of the spreading model, because the stochastic spreading of heterochromatin is often discussed in an overtly simplified scenario where cells are considered homogenous in their ability (probability) to silence. This result, based on the PEV eye phenotype, clearly points to cell-to-cell variation in the probability of reporter silencing that is consistent across individual flies. In other words, while the random spreading model (i.e., equal probability of spreading across all cells) is adequate for explaining sectors of PEV reporter expression in *S. pombe* colonies [1, 5, 12], in higher eukaryotes, it appears that a more elaborate model considering developmental lineage or environmental differences between cells displaying the phenotype is needed to adequately describe the process. Consistent with this conjecture, we did not find the Y chromosome BL2 reporter, which is in a different *cis*-regulatory environment and likely subject to different regulation during the process of developmental differentiation, to express similar patterns in the eye as the 39C-12 reporter, despite having been transferred into the same A1 or D1 genetic background. One suggestion, amongst many other possibilities, as to the source of differences between cells in their probability of heterochromatin silencing is the timing of the last wave of cell divisions during metamorphosis. Using the beta-gal reporter, Lu et al. [26] reported that in the eye disk, there is a dramatic difference in variegation on either side of the morphogenetic furrow, with a relaxation of silencing at this juncture. Such timing differences between geographical locations of the eye could be a contributor, as cells that go through an S phase later in developmental time are inherently exposed to a different environment during the silencing/relaxation process.

## Conclusions

In summary, our observations with two PEV reporters inserted in different *cis*-regulatory environments in two diverging *trans*-acting genetic backgrounds (A1 and D1) indicate that while *trans*-acting modifiers play the major role in determining the degree of PEV silencing, the pattern of PEV silencing is likely influenced more by the *cis*-regulatory environment of the reporter insertion site.

## Materials and methods

### Fly husbandry and genetics

Flies were cultured at 25 °C, 70% humidity on regular cornmeal sucrose-based medium [29]. Unless otherwise specified, genetic crosses were performed by mating two male flies with three–five female virgin flies. The 39C-12 reporter line [13] was used as the starting line to generate A1 and D1 inbred lines. Five generations of consecutive full sibling crosses with selection for extreme eye phenotype at each generation were performed to create the two inbred lines. To substitute the BL2 Y-linked PEV reporter [23] into the A1 or D1 genetic background, dominantly marked balancers were used to follow the second and third chromosomes (see Additional file 2: Figure S2 for crossing scheme). Balancers SM5 and TM6 were first introduced to the BL2 line by a standard cross, and the F1 male progeny that had both the second and third chromosomes balanced were selected (based on the dominant markers) to mate with female flies from the inbred line. A male F2 progeny from the F1 cross with both balancers over inbred chromosomes were selected to backcross to three–five inbred line female virgins. Only one male fly was used in the F2 cross to ensure that there would only be two genotypes of the 4th chromosome in the F3 population (i.e., the original 39C-12 4th chromosome and the other 4th chromosome, which is denoted as +^iso^ in Additional file 2: Figure S2, carried by the selected F2 male). Because the 4th chromosome is not known to recombine during meiosis (or does so extremely rarely), progeny from the backcross lacking both balancers was selected to make a floating stock (i.e., inbred genetic background with an unmarked 4th chromosome floating). This floating stock was made homozygous for the 39C-12 4th chromosome, as judged by the presence of the 39C-12 reporter expression, to generate the final stock. To evaluate the homozygosity of the 4th chromosome in the final stock, 39C-12 reporter expression in all female progeny was followed by visual inspection for multiple generations (*white* expression in male progeny cannot be used to evaluate 39C-12 expression because of the interference coming from the mini-*white* construct in the BL2 reporter.)

### PEV assays

Eye pigment extraction and quantification was done essentially as previously described [30] with a few modifications. Instead of hand homogenizing for pigment extraction, whole flies were homogenized using a Mixer Mill Mm 300 to increase the throughput and consistency. The overnight incubation at 4 °C was omitted. For each genotype of each sex, 20–30 age-matched flies (3–5 days old) were randomly selected from the population and sorted according to their pigmentation level by visual inspection. Five flies of similar pigmentation levels were then collected together as one sample. The same protocol was used for both sexes.

X-gal staining of eye imaginal discs and the assay of *beta*-galactosidase activity were carried out as previously described [31].

For image analysis of the PEV phenotype, eye pictures from a random sampling of the PEV phenotype from each population were selected based on eye size and angle of the photo to ensure consistent quantification. Each image was then converted to 8-bit gray scale in imageJ, and then further converted to a binary image through manual threshold setting. The guideline used for threshold setting was to capture as many variegating speckles as possible without introducing large patches of shadow that resulted from lighting differences during imaging. The area to quantify was manually selected using the imageJ *oval tool* to cover as much of the eye as possible without covering other anatomical structures. The binary oval image representation of the eye PEV phenotype was then converted into a binary table of quantification using the imageJ *image to result* function, where each entry in the table represents a pixel in the image. To test for enrichment of pigmentation in the ventral-posterior quadrant, the proportion of “expressed” pixels in the ventral-posterior quadrant was compared to the proportion of “expressed” pixels outside of the quadrant of interest using statistical tests described in the following section.

### Statistical analysis

Analysis of the strength of the PEV phenotype (e.g., pigment level) was done using either *R* or excel. For estimating the genotype effect and the sex effect on the variation in PEV phenotype between the A1 and D1 lines, we fit the pigment assay data to a linear model using the genotype label and sex label as predictors. More precisely, the OD 480 reading for pigment level was first log transformed and then fitted as the response variable in the following linear model using the lm() function in *R*:

$$P_{i} = \mu + \beta_{1} G_{i} + \beta_{2} S_{i} + \varepsilon_{i}$$

where *P*_*i*_ represents the pigment level of individual *i*, *G*_*i*_ is the indicator variable for the genotype label, and *S*_*i*_ is the indicator variable for the sex label. The adjusted *R*^2^ produced by applying the summary() function to the above-described lm object is used to estimate the percentage variance explained by the model. To evaluate the significance of the genotype effect (i.e., the differences in PEV phenotype between the A1 and D1 genotypes), we performed an *F* test by applying the anova() function on the lm object. Analysis of the pattern of PEV followed a similar linear model framework, where the proportion of pigmentation in or out of the ventral-posterior quadrant is modeled using a binary predictor of location. To evaluate the association between the proportion of pigmentation and the location, we performed an *F* test.

## Supplementary information

**Additional file 1: Figure S1.** Differences in the coefficient of variation (CV) between groups are not driven by extreme samples. Standard Boxplots are shown summarizing the range of CVs calculated for each group based on CVs of all permutations of leaving one sample out. The outliers for boxplots are defined as data points that lie beyond plus/minus 1.5 times the inter quartile range from the top/bottom quartile. Note that in any given combination of the leave-one-out CV values, the 39C12 starting line and the F2 population have consistently higher CV values than the inbred populations.

**Additional file 2: Figure S2.** Crossing scheme for creating isogenic BL2 reporter lines. The BL2 reporter on the Y chromosome was first crossed into a balancer stock to recover the second and third chromosome dominant markers. The F1 male progeny with second and third chromosomes dominantly marked were selected to cross with female virgins of the A1 inbred line. A single F2 male progeny (blue rectangle) was selected to back cross to 3~5 A1 inbred line female virgins. The F3 progeny that have no balancer chromosomes will have the BL2 reporter in the A1 background with a non-A1 4th chromosome floating in the population. Note that the non-A1 4th chromosome was introduced from a single F2 male, which means that in the F3 population there are only two genotypes of the 4th chromosome (denoted 39C-12 and +^iso^ respectively). The F3 virgin flies that have no balancer chromosomes were selected to create a floating stock. In order to separate the 39C-12 chromosome from +^iso^ chromosome, single sibling pairs from the F3 floating stock were isolated to create multiple stocks; the 39C-12 reporter expression in all female fly eyes was followed by visual inspection for several generations in order to identify a homozygous 39C-12 stock (i.e. the exact A1 background with the Y chromosome containing the BL2 reporter). The same approach was used to transfer the BL2 reporter to the D1 genetic background.

**Additional file 3: Figure S3.** Example images to illustrate the processing steps for quantifying the pattern of PEV. (A) The original photo of a representative variegating eye phenotype taken from an F1 male progeny of an A1 by D1 cross. (B) An 8-bit grey scale version of A transformed using imageJ. (C) A binary image of B generated using imageJ. The image was first rotated so that the maximal area of the fly eye could be selected using the oval tool. Pixels outside the selected oval area were removed (pseudo-colored in grey for illustration) while pixels within the oval area were converted to binary by setting a threshold. The threshold selection was done manually to best represent the original eye phenotype. To evaluate the similarity of the PEV pattern between individuals, each image of a fly eye was split into four even quadrants (blue lines) and the pigment enrichment (i.e. the proportion of black pixels) in the ventral-posterior quadrant (red arrow) was evaluated against the area outside the ventral-posterior quadrant.

## Data Availability

The data used and/or analyzed during the current study are available from the corresponding author on reasonable request.
