# Supplementary figures and images for: The impact of genetic background and cell lineage on the level and pattern of gene expression in position effect variegation
Source: Epigenetics Chromatin. 2019 Nov 13;12:70. doi: 10.1186/s13072-019-0314-5 (PMC6852933; doi:10.1186/s13072-019-0314-5)

Figure S1

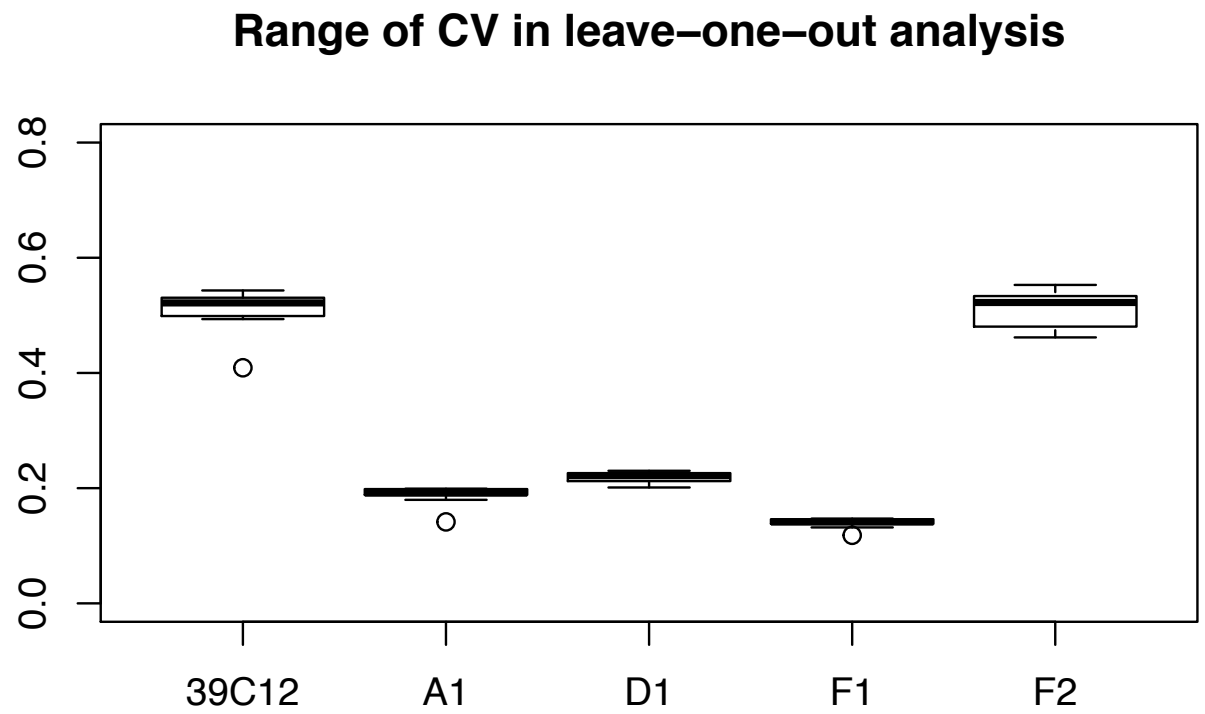

Supplement: Supplementary file 1 — Additional file 1: Figure S1. Differences in the coefficient of variation (CV) between groups are not driven by extreme samples. Standard Boxplots are shown summarizing the range of CVs calculated for each group based on CVs of all permutations of leaving one sample out. The outliers for boxplots are defined as data points that lie beyond plus/minus 1.5 times the inter quartile range from the top/bottom quartile. Note that in any given combination of the leave-one-out CV values, the 39C12 starting line and the F2 population have consistently higher CV values than the inbred populations. [file 13072_2019_314_MOESM1_ESM.pdf]

Figure S2

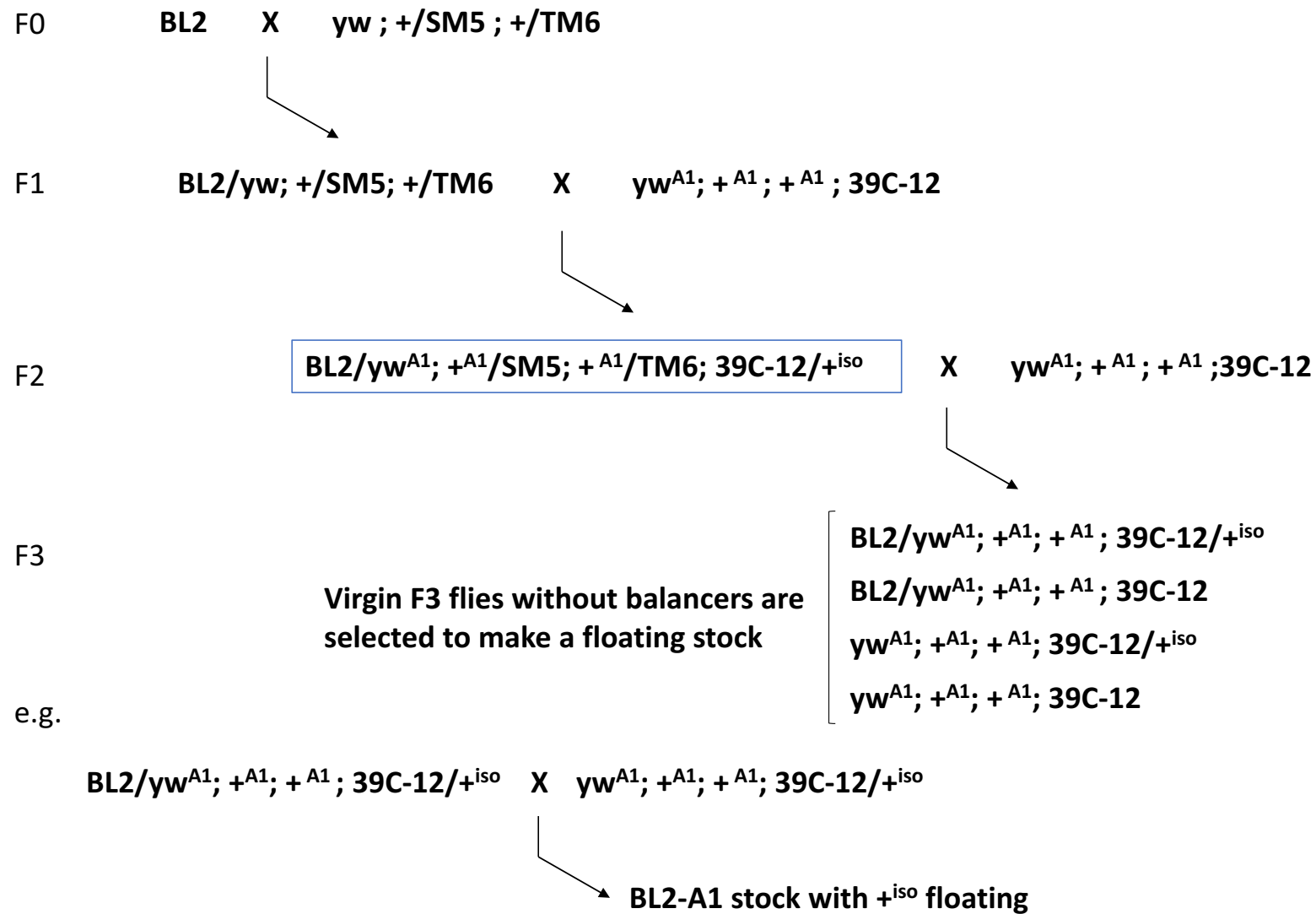

Supplement: Supplementary file 2 — Additional file 2: Figure S2. Crossing scheme for creating isogenic BL2 reporter lines. The BL2 reporter on the Y chromosome was first crossed into a balancer stock to recover the second and third chromosome dominant markers. The F1 male progeny with second and third chromosomes dominantly marked were selected to cross with female virgins of the A1 inbred line. A single F2 male progeny (blue rectangle) was selected to back cross to 3~5 A1 inbred line female virgins. The F3 progeny that have no balancer chromosomes will have the BL2 reporter in the A1 background with a non-A1 4th chromosome floating in the population. Note that the non-A1 4th chromosome was introduced from a single F2 male, which means that in the F3 population there are only two genotypes of the 4th chromosome (denoted 39C-12 and +iso respectively). The F3 virgin flies that have no balancer chromosomes were selected to create a floating stock. In order to separate the 39C-12 chromosome from +iso chromosome, single sibling pairs from the F3 floating stock were isolated to create multiple stocks; the 39C-12 reporter expression in all female fly eyes was followed by visual inspection for several generations in order to identify a homozygous 39C-12 stock (i.e. the exact A1 background with the Y chromosome containing the BL2 reporter). The same approach was used to transfer the BL2 reporter to the D1 genetic background. [file 13072_2019_314_MOESM2_ESM.pdf]

Figure S3

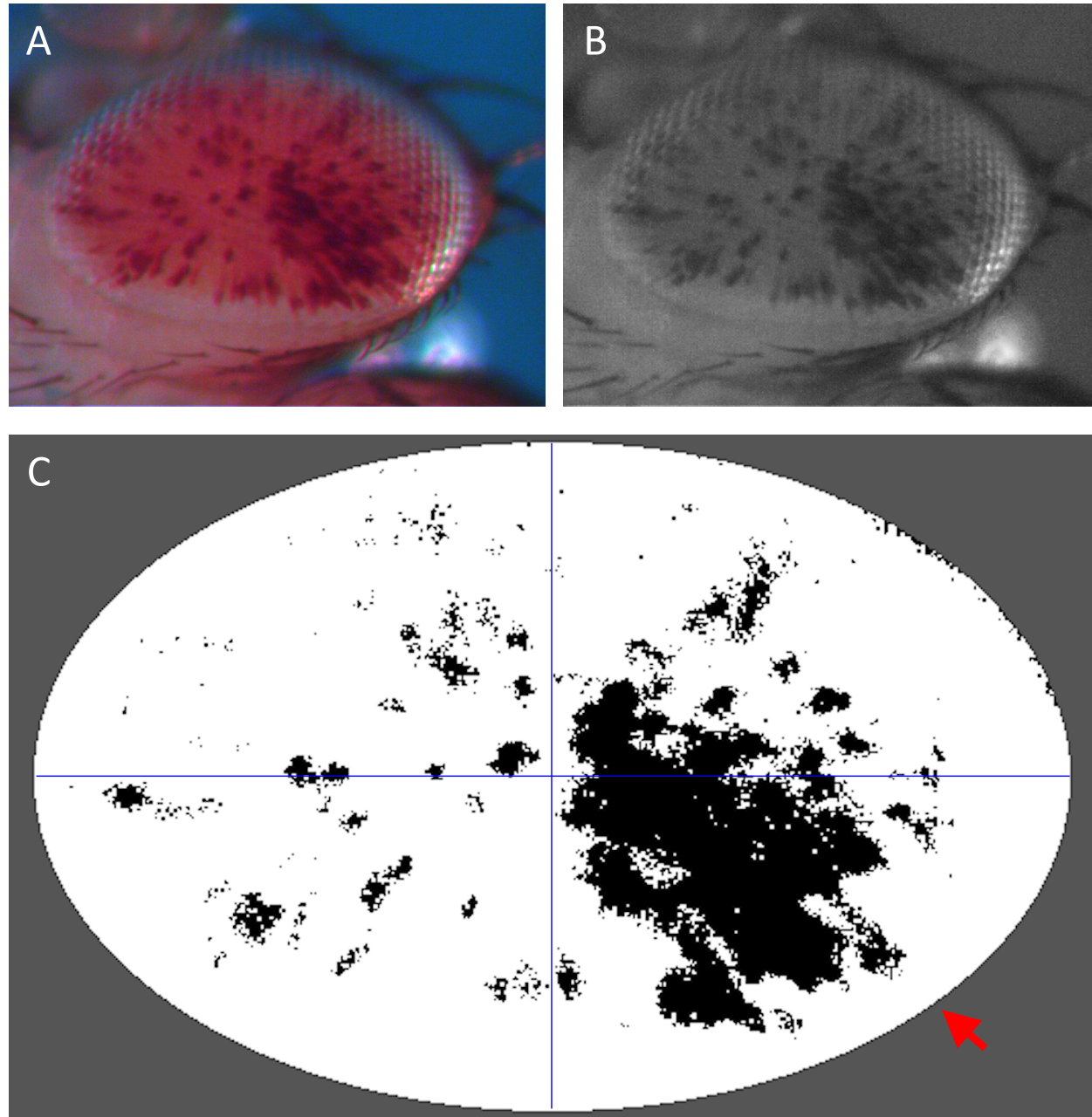

Supplement: Supplementary file 3 — Additional file 3: Figure S3. Example images to illustrate the processing steps for quantifying the pattern of PEV. (A) The original photo of a representative variegating eye phenotype taken from an F1 male progeny of an A1 by D1 cross. (B) An 8-bit grey scale version of A transformed using imageJ. (C) A binary image of B generated using imageJ. The image was first rotated so that the maximal area of the fly eye could be selected using the oval tool. Pixels outside the selected oval area were removed (pseudo-colored in grey for illustration) while pixels within the oval area were converted to binary by setting a threshold. The threshold selection was done manually to best represent the original eye phenotype. To evaluate the similarity of the PEV pattern between individuals, each image of a fly eye was split into four even quadrants (blue lines) and the pigment enrichment (i.e. the proportion of black pixels) in the ventral-posterior quadrant (red arrow) was evaluated against the area outside the ventral-posterior quadrant. [file 13072_2019_314_MOESM3_ESM.pdf]
